# A prospective cohort study in depression and anxiety among Vietnamese migrants in Japan during the early to mid-COVID-19 pandemic

**DOI:** 10.1186/s41182-024-00605-4

**Published:** 2024-07-01

**Authors:** Tadashi Yamashita, Pham Nguyen Quy, Chika Yamada, Emi Nogami, Kenji Kato

**Affiliations:** 1 Faculty of Nursing, Kobe City College of Nursing, 3-4 Gakuennishi-machi, Nishi-ku, Kobe, Hyogo 651-2103 Japan; 2 Department of Medical Oncology, Kyoto Miniren Central Hospital, 2-1 Uzumasa Tsuchimoto-cho, Ukyo-ku, Kyoto, 616-8147 Japan; 3 Department of Environmental Coexistence, Center for Southeast Asian Studies, Kyoto University, 46 Shimoadachi-cho, Yoshida Sakyo-ku, Kyoto, 606-8501 Japan; 4 Department of Social Welfare, School of Psychology and Social Welfare, Mukogawa Women’s University, 6-46, Ikebiraki, Nishinomiya, Hyogo 663-8558 Japan; 5 Faculty of Nursing, Kobe Women’s University, 4-7-2, Minatojima Nakamachi, Chuo-ku, Kobe, Hyogo 650-0046 Japan

**Keywords:** COVID-19, Vietnam in Japan, Migrants, Depression, Anxiety disorder, Changes in socioeconomic status, Having someone with whom to discuss one’s health

## Abstract

**Introduction:**

The enduring COVID-19 pandemic has had persistent, intermittent socioeconomic impacts on migrants. This raises the concern that many Vietnamese migrants in Japan may have developed mental health issues due to the socioeconomic impact. The study aimed to examine changes in the socio-economic and mental health status of Vietnamese migrants in Japan and factors affecting mental health status during the early to mid-COVID-19 period.

**Methods:**

We conducted a prospective cohort study among Vietnamese migrants in Japan from September to October 2021 (baseline) and from May to June 2022 (follow-up) using an online questionnaire. Multiple linear regression analyses were conducted to examine the association between changes in socioeconomic status and alterations in symptoms of depression and anxiety within this demographic.

**Results:**

The mean age of the 159 participants was 26.1 ± 4.9 years, with a mean length of residency in Japan of 4.0 ± 4.1 years. The mean PHQ-9 score exhibited a significant decrease from 7.89 (*SD* = 6.34) to 6.62 (*SD* = 5.87) (*p* = 0.01). Variables associated with changes in depression and anxiety included subjective socioeconomic status (unstandardized partial regression coefficient (UPRC): 1.901, 95% confidence interval (CI) 0.30 to 3.50, p = 0.02) and (UPRC: 2.060, 95% CI 0.80 to 3.32, p = 0.002), as well as changes in having someone with whom to discuss one’s health (UPRC: 2.689, 95% CI 0.89 to 4.49, p = 0.004) and (UPRC: 1.955, 95% CI 0.54 to 3.38, p = 0.007).

**Conclusions:**

In this prospective cohort study of depression and anxiety, depressive symptoms among Vietnamese migrants decreased from 2021 to 2022. Key findings underscore the importance of socioeconomic status improvement and having someone to discuss to about their health as protective factors against mental health challenges. Employment and social support have emerged as crucial determinants of mental health among Vietnamese migrants in Japan, emphasizing the necessity for comprehensive support strategies addressing both economic vulnerabilities and social connectedness.

## Background

Migrants are highly susceptible to depression due to the stress of adjusting to a new life [1]. The World Health Organization (WHO) has documented the considerable adverse effects of the COVID-19 pandemic on the socioeconomic circumstances of refugees and migrants [2]. COVID-19 could worsen migrants’ mental health by exacerbating their already unstable economic and social situations. A comprehensive review reported that COVID-19 limited migrants' ability to cope with the socio-psychological aspects of their lives, in addition to limiting their ability to avoid infectious diseases and access to healthcare service [3]. Hence, there exists apprehension regarding the ramifications of the COVID-19 pandemic on the mental health of migrant populations. The living conditions experienced by migrants are notably diverse, contingent upon their country of origin, socioeconomic status, and prevailing conditions in the destination country. Therefore, elucidating the impact of the COVID-19 pandemic on the mental health of migrants residing in Japan necessitates focused investigation. Nonetheless, research delineating the effects of the COVID-19 pandemic on the mental health of migrants in Japan remains exceedingly scarce.

The number of migrants has significantly increased in the past decade in Japan, attributed to an increase in the number of foreign technical interns and students [4]. Previous study focusing on foreign technical interns in Japan suggest that they are at higher risk of mental health disruption [5]. Moreover, a cross-sectional study conducted in 2021 of foreign care workers in Japan’s long-term care facilities revealed a marginally inferior mental health status among foreign care workers compared to their Japanese counterparts [6]. The Vietnamese community is the second-largest migrant group in Japan, exhibiting accelerated growth relative to other nationalities. Nonetheless, multiple reports underscore their confrontation with an array of health adversities. For instance, Vietnamese technical interns reported impediments in seeking medical assistance until their symptoms exacerbated [7]. The study also discerned a deterioration in female-specific physical conditions among Vietnamese technical interns, compounded by the exacerbation of symptoms attributable to their reluctance to communicate their ailments [8]. Uezato et al. conducted a cross-sectional survey in 2022 and reported that Vietnamese migrants in Japan may be suffering through their interactions with Japanese colleagues as part of their cultural adjustment to Japan [9]. Our group also investigated a cross-sectional study to determine the actual changes in the lifestyle and mental well-being among Vietnamese living in Japan as of 2021, the nascent phase of the COVID-19 pandemic. The findings indicated that roughly 70% of Vietnamese migrants in Japan encountered a decline in their income compared to pre-pandemic levels [10]. These revelations intimate that certain Vietnamese migrants might have encountered restricted access to medical services, healthcare obstacles, psychological distress stemming from cultural adaptation to Japan, and even financial deprivation due to the COVID-19 pandemic. However, there are no studies investigate the changes in mental health issues among Vietnamese migrants in Japan throughout the enduring COVID-19 pandemic.

Research on mental health during the COVID-19 pandemic is very limited. A survey of 3 million U.S. adults conducted between April 2020 and August 2022 found that levels of anxiety and depression were highest in late 2020, followed by anxiety and depression beginning to decline in early 2021 [11]. In addition, a longitudinal study of mental health in the Japanese population in 2021 reported that the number of people who were worried about COVID-19 infection decreased from April to June 2021 to October to December 2021 [12]. However, there are no studies that quantitatively evaluate the mental health status of migrants living in Japan. It would be important to investigate the mental health status of Vietnamese migrants in Japan, to consider interventional measures for migrants in Japan as a whole.

To fill this knowledge gap, we conducted a prospective cohort study of their health status in Japan. The study aimed to examine changes in the socio-economic and mental health status of Vietnamese migrants in Japan and factors affecting mental health status during the early to mid-COVID-19 period. Knowing whether depression and anxiety symptoms among Vietnamese migrants in Japan persisted throughout multiple years during the COVID-19 pandemic and identifying associated factors would help guide health policy for migrants.

## Methods

**Fig. 1:**
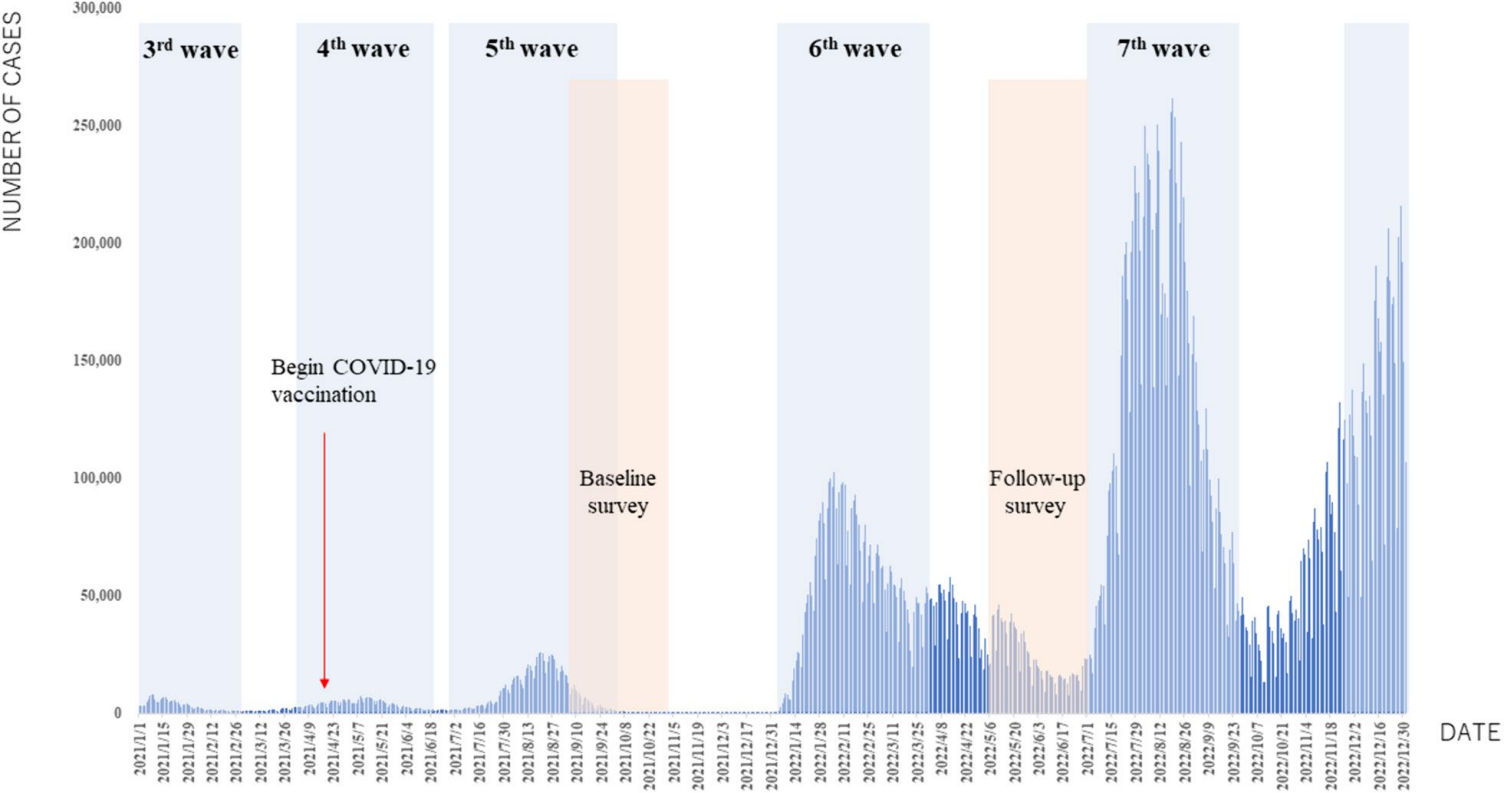
Trends of COVID-19 cases in Japan from the third to seventh waves and the commencement of the COVID-19 vaccination campaign

We conducted a prospective cohort study among Vietnamese migrants in Japan from September to October 2021 (baseline) and from May to June 2022 (follow-up). In Japan, the baseline survey period coincided with the fifth wave of the COVID-19 pandemic, while the follow-up period occurred between the sixth and seventh waves of the pandemic. During the follow-up period, there was an increase in the number of COVID-19 cases compared to the baseline period. Figure 1 illustrates the trends of COVID-19 cases in Japan, spanning from the third to seventh waves of the pandemic, along with the commencement of the COVID-19 vaccination campaign.

A total of 1046 individuals were invited to participate in this baseline survey, with 621 individuals included in the analysis. Among the 167 individuals who took part in both the baseline and follow-up surveys, eight participants with incomplete responses were excluded. Consequently, a total of 159 participants were included in the analysis. Eligibility criteria for participation required individuals to hold Vietnamese or Japanese nationality, be born in Vietnam, and be at least 18 years of age.

### Data collection tools

We utilized the web-based survey tool SurveyMonkey (Momentive Inc., San Mateo, CA, USA) for data collection. Leaflets were distributed at churches and on Facebook, the primary social media platform used by the Vietnamese community in Japan. These leaflets provided comprehensive information regarding the study’s background, objectives, methodology, and ethical considerations. Additionally, we encouraged participation through a community group supported by Vietnamese, which is frequented by many Vietnamese migrants in Japan. The online questionnaire, administered entirely in Vietnamese, collected demographic information (gender, age, duration of residence in Japan, marital status, education, residential status, Japanese language proficiency, and diseases under treatment), as well as social and economic data (health insurance, subjective socioeconomic status, connections with the Vietnamese community in Japan, and availability of having someone with whom to discuss one’s health). The questions assessing Japanese language proficiency were based on the Japanese Language Proficiency Test, mandated for certain residential statuses, employing a four-question format developed by the co-researchers with input from a language specialist. Clinical depression was evaluated using the Patient Health Questionnaire-9 (PHQ-9), and symptoms of anxiety were assessed using the Generalized Anxiety Disorder 7-item (GAD-7) scale. Both instruments have been validated for use among migrant populations and the Vietnamese community [13–15]. Responses to the PHQ-9 and GAD-7 items were recorded on a 4-point Likert scale (0–3), resulting in scores ranging from 0 to 27 for the PHQ-9 and from 0 to 21 for the GAD-7. Cut-off scores for the PHQ-9 and GAD-7 were defined as ≥ 5, ≥ 10, ≥ 15, and ≥ 20 for mild, moderate, moderately severe, and severe depression, respectively [14, 16], and as ≥ 5, ≥ 10, and ≥ 15 for mild, moderate, and severe anxiety disorder, respectively [17, 18]. Clinically significant conditions were indicated by scores of ≥ 10 on the PHQ-9 and ≥ 5 on the GAD-7 for Vietnamese individuals [19–21].

### Statistical analysis

Descriptive analysis utilized means and standard deviations (SDs) for continuous variables, and counts and percentages for categorical variables. Due to the non-normal distribution of total scores on the PHQ-9 and GAD-7, the Wilcoxon signed rank test was employed to compare differences between baseline and follow-up. Additionally, both univariate and multiple linear regression analyses were performed to identify factors influencing depression and anxiety disorders. The dependent variable for both PHQ-9 and GAD-7 was the difference between baseline and follow-up, with baseline serving as the reference year. The independent variables included subjective socioeconomic status and having someone with whom to discuss one’s health. Subjective socioeconomic status and availability of having someone with whom to discuss one’s health were categorized as follows: + 1 for improvement, 0 for no change, and − 1 for deterioration at follow-up. Confounding factors for the multiple linear regression analyses were age, gender, length of stay in Japan, and educational background, referring to a systematic review article on the mental health of foreign residents in Japan [22]. We confirmed that the multiple linear regression model finally selected was equivariance by residual analysis. To assess multicollinearity, the Variance Inflation Factor between independent variables was ensured to be less than 10. Finally, to evaluate dropouts from the follow-up survey, independent samples t-tests, chi-square tests, and Fisher exact tests were utilized to compare differences between respondents and dropouts from baseline. Statistical analyses were performed using SPSS software (version 26.0; IBM Corp., Armonk, NY, USA), with a significance level of p < 0.05 set for two-sided tests.

### Ethical consideration

The current study (Approval number: 20124-05) received approval from the ethical committee of Kobe City College of Nursing, and all participants provided consent in compliance with the Declaration of Helsinki.

## Results

The mean age of the participants was 26.1 ± 4.9 years, with a mean length of residence in Japan of 4.0 ± 4.1 years. Out of the participants, 82 (51.6%) were male and 77 (48.4%) were female. The most common educational background was university education 60 (37.7%), following high school education 51 (32.1%). In terms of Japanese language proficiency, 84 (52.8%) participants could speak enough to not affect their work or study, 41 (25.8%) participants could speak enough to not affect their daily life, 23 (14.5%) participants could speak fluently, and 11 (6.9%) participants could barely speak Japanese. Additionally, 59 (37.1%) reported having someone with whom to discuss one’s health. Regarding subjective socioeconomic status, 70 (44.0%) participants reported” slightly difficult”, 55 (34.6%) participants reported “general”, 17 (10.7%) reported “very difficult” and 16 (10.1%) reported “slightly better”. Comparing participants with dropouts from the follow-up survey, no statistically significant associations were found for age (*p* = 0.672), duration of residence in Japan (*p* = 0.193), gender (*p* = 0.165), PHQ-9 (*p *= 0.909), and GAD-7 (*p* = 0.091). Items with statistically significant associations between dropouts and participants were Japanese language level (*p* = 0.007) and availability of having someone with whom to discuss one’s health (*p* = 0.021) (Table 1).

**Table 1:** Socio-demographic characteristics, PHQ-9 and GAD-7 scores among Vietnam migrants in Japan of baselines and dropouts, respectively

Items	Baselines (n = 159)		Dropouts (n = 462)		
	n	%	n	%	p value
Age					
Mean ± SD	26.1 ± 4.9		25.9 ± 4.5		0.672
Duration of residence in Japan					
Mean ± SD	4.0 ± 4.1		3.7 ± 3.2		0.193
Gender					
Male	82	51.6	266	57.6	0.165
Female	77	48.4	196	42.4	
Educational background					
Junior High school	0	0	5	1.1	0.075
High school	51	32.1	186	40.3	
Technical school	33	20.8	100	21.6	
College	3	1.9	11	2.4	
University	60	37.7	141	30.5	
Graduate school	12	7.5	19	4.1	
Japanese language level					
Ability to speak fluently	23	14.5	51	11.0	0.007
Ability to speak enough to not affect their work or study	84	52.8	187	40.5	
Ability to speak enough to not affect their daily life	41	25.8	173	37.4	
Barely able to speak Japanese	11	6.9	51	11.0	
Having someone with whom to discuss one’s health					
Have	59	37.1	128	27.7	0.021
Do not have	100	62.9	334	72.3	
Subjective socioeconomic status					
Really good	1	0.6	4	0.9	0.151
Slightly better	16	10.1	31	6.7	
General	55	34.6	135	29.2	
Slightly difficult	70	44.0	222	48.1	
Very difficult	17	10.7	70	15.2	
PHQ-9					
Mean ± SD	8.7 ± 5.7		8.8 ± 5.6		0.909
GAD-7					
Mean ± SD	4.8 ± 5.1		5.6 ± 5.5		0.091

Compared to the baseline survey, 62 (39.0%) had improved their subjective socioeconomic status in the follow-up survey, 36 (22.6%) had worsened, and 61 (38.4%) had not changed. And, compared to the baseline survey, 40 (25.2%) had improved in the follow-up survey, 29 (18.2%) had worsened, and 90 (56.6%) had no change in having someone with whom to discuss one’s health.

**Fig. 2:**
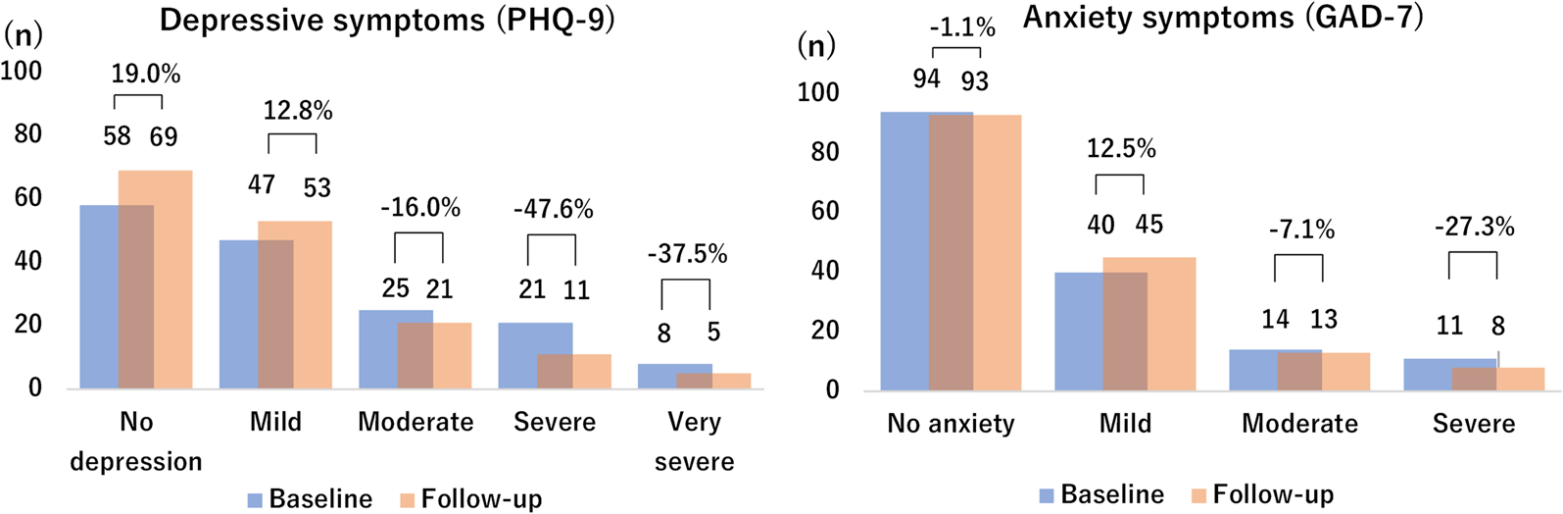
Changes of depression and anxiety symptoms from baseline to follow-up

The PHQ-9 score significantly decreased during our study period (7.89 ± 6.34 vs. 6.62 ± 5.87, *p* = 0.01). The severity of depression symptoms also decreased, with a 16.0% decrease in “moderate”, a 47.6% decrease in “severe”, and a 37.5% decrease in “very severe” symptoms. The GAD-7 score showed a slight decrease from 4.80 ± 5.19 at baseline to 4.53 ± 4.84 at the follow-up survey, although this difference was not statistically significant (*p* = 0.589). However, the severity of anxiety symptoms also decreased with time, with a 7.1% decrease in “moderate,” and a 27.3% decrease in “severe” symptoms (Fig. 2).

In the multiple linear regression analysis model, age (unstandardized partial regression coefficient (UPRC): − 0.356, 95% confidence interval (CI) − 0.66 to − 0.05, *p* = 0.021), changes in subjective socioeconomic status (UPRC: 1.901, 95% confidence interval (CI) 0.30 to 3.50, *p* = 0.02), and changes in having someone with whom to discuss one’s health (UPRC: 2.689, 95% CI 0.89 to 4.49, *p* = 0.004), were found to be associated with symptoms of depression (Table 2). In addition, age (UPRC: − 0.276, 95% CI − 0.51 to − 0.04, *p* = 0.024), changes in subjective socioeconomic status, (UPRC: 2.060, 95% CI 0.80 to 3.32, *p* = 0.002), and changes in having someone with whom to discuss one’s health (UPRC: 1.955, 95% CI 0.54 to 3.38, *p* = 0.007) were found to be associated with symptoms of anxiety (Table 3).

**Table 2:** Multiple linear regression analysis of the difference of the PHQ-9 scores between baseline and follow-up, with baseline as the reference year (n = 159)

	Crude model		Adjusted model*	
	Unstandardized coefficients [95% CI]	*p*-value	Unstandardized coefficients [95% CI]	*p*-value
Age	− 0.466 [− 0.71 to − 0.23]	< 0.001	− 0.356 [− 0.66 to − 0.05]	0.021
Gender	0.495 [− 1.94 to 2.93]	0.689	0.626 [− 1.70 to 2.94]	0.595
Duration of residence in Japan	− 0.383 [− 0.68 to -0.09]	0.011	− 0.020 [− 0.36 to 0.32]	0.907
Education level	0.763 [− 0.62 to 2.15]	0.278	− 0.669 [− 2.08 to 0.74]	0.351
Changes in subjective socioeconomic status	2.817 [1.29 to 4.34]	< 0.001	1.901 [0.30 to 3.50]	0.02
Changes of having someone with whom to discuss one’s health	3.504 [1.73 to 5.28]	< 0.001	2.689 [0.89 to 4.49]	0.004

* Adjusted model: Adjusted for baseline score of age, gender, duration of residence in Japan, and education level

CI: confidence interval

**Table 3:** Multiple linear regression analysis of the difference of the GAD-7 scores between baseline and follow-up, with baseline as the reference year (n = 159)

	Crude model		Adjusted model*	
	Unstandardized coefficients [95% CI]	*p*-value	Unstandardized coefficients [95% CI]	*p*-value
Age	− 0.336 [− 0.53 to − 0.14]	< 0.001	− 0.276 [− 0.51 to − 0.04]	0.024
Gender	0.697 [− 1.23 to 2.63]	0.477	0.848 [− 0.98 to 2.68]	0.361
Duration of residence in Japan	− 0.193 [− 0.43 to 0.04]	0.108	0.121 [− 0.15 to 0.39]	0.376
Education level	0.844 [− 0.25 to 1.94]	0.129	− 0.301 [− 1.42 to 0.81]	0.594
Changes in subjective socioeconomic status	2.604 [1.42 to 3.79]	< 0.001	2.060 [0.80 to 3.32]	0.002
Changes of having someone with whom to discuss one’s health	2.668 [1.26 to 4.08]	< 0.001	1.955 [0.54 to 3.38]	0.007

* Adjusted model: Adjusted for baseline score of age, gender, duration of residence in Japan, and education level

CI: confidence interval

## Discussion

The number of COVID-19 patients in Japan increased rapidly from 2021 to 2022, whereas within this study, there was a progressive decline observed in depressive symptoms among Vietnamese migrants in Japan during the same period. Our findings reveal a positive association between amelioration in subjective economic circumstances and the presence of confidants for health-related discussions, and a reduction in depressive and anxiety symptoms among Vietnamese migrants in Japan. These findings underscore the imperative of not only bolstering the socioeconomic well-being of Vietnamese migrants in Japan during the COVID-19 crisis but also instituting frameworks to cater to their mental health in prospective pandemics.

This study observed a statistically significant decline in depressive symptom scores among Vietnamese residents in Japan from 2021 to 2022. Additionally, there was a reduction in anxiety symptoms, albeit not statistically significant. Psychological stress among full-time workers in Japan, including migrant workers deteriorated during the second wave of the COVID-19 pandemic in August 2020, but subsequently stabilized and has been improving since October 2021 [23]. Furthermore, a longitudinal study examining participants aged 20 to 79 years who lived in Japan, including migrants, between 2020 and 2022 reported that levels of depression increased in the early stages of the pandemic and decreased in January 2022 [24]. In Japan, the number of COVID-19 cases surged from March to June 2022 (follow-up survey period in this study), between the sixth and seventh waves, compared to the fifth wave of COVID-19 cases that occurred from June to October 2021 (baseline survey period in this study). Conversely, the availability of the COVID-19 vaccine to the Japanese population since April 2021 may have mitigated some apprehensions among residents, including migrants. As for the situation of Vietnamese migrants in Japan, it was very difficult for them to return to Vietnam from Japan between 2020 and 2021, as the Vietnamese government was taking measures to prevent infection. Consequently, Vietnamese migrants in Japan were predisposed to feelings of profound loneliness and anxiety. However, in 2022, the Vietnamese government revised the policy to coexist with the virus, and Vietnamese migrants in Japan can now return to their home country. It is plausible that this relaxation in repatriation restrictions may have influenced the mental health of Vietnamese migrants in Japan.

The study identified a positive correlation between improvement in subjective economic conditions and a decrease in depression and anxiety symptoms among Vietnamese migrants in Japan amid the COVID-19 pandemic. Employment emerged as a significant factor safeguarding mental health, particularly concerning depression and psychological distress [24]. A study conducted in Japan during the COVID-19 pandemic reported that among participants who lived in Japan, including migrants, having suffered an economic impact was one risk factor for depressive symptoms [25]. These findings suggest that the economic impact caused by the COVID-19 pandemic could have had a significant impact on the mental health of migrants in Japan. Given the economic vulnerability often observed among migrants, it is a valid concern that the economic situation indicated by this study has an impact on the mental health of Vietnamese migrants in Japan. Given that employment serves as a protective determinant for the psychological welfare of Vietnamese migrants in Japan, it is imperative to direct attention towards the economic susceptibilities of this demographic, along with devising methodologies to intertwine economic support initiatives with their mental health welfare.

The multiple linear regression analysis in this study revealed a connection between the availability of having someone with whom to discuss their health for Vietnamese migrants and their mental health amid the COVID-19 pandemic. People with whom one can discuss their health are family members, close friends, and health professionals at neighborhood clinics and health centers. Research indicates that individuals with friends and confidants tend to experience greater life satisfaction and a reduced likelihood of depression [26]. On the other hand, health disparities have been observed between migrants in Japan and the Japanese population due to linguistic barriers and differences in the medical system from their home countries [27]. Moreover, low rates of mental health consultation among migrants in Japan suggest limited access to mental health counseling [28]. Considering these factors, the availability of opportunities for Vietnamese migrants in Japan to discuss their health may mitigate stress accumulation among them, potentially preventing mental health issues. This significance is further underscored during periods of societal disruption such as the COVID-19 pandemic.

The prospective cohort study on Vietnamese migrants in Japan observed a decline in their mental health status from 2021 to 2022 and identified several factors associated with these changes during the COVID-19 pandemic. However, the study had several research limitations that may make it difficult to generalize to the entire Vietnamese population in Japan. These limitations included a small sample size, subjective responses as variables, and self-reported data, such as nationality. Additionally, approximately 40% of the participants held a university degree as their highest level of education, potentially indicating higher education levels compared to the broader Vietnamese migrant population in Japan. Research has indicated a correlation between years of education and decreased symptoms of depression and anxiety [29]. This study may have underestimated the mental health status of Vietnamese migrants in Japan. Moreover, some participants dropped out between baseline and follow-up survey, potentially introducing bias. Although no significant differences in depression or anxiety were observed between participants and dropouts, the analysis may have overestimated certain variables due to the dropout of participants with lower proficiency in Japanese language and fewer opportunities for discussing their health with others. Additionally, some study participants arrived in Japan after the onset of the COVID-19 pandemic, suggesting potential variations in pandemic-related concerns among participants. Nonetheless, the study offers valuable insights into the mental health status of Vietnamese migrants in Japan during the pandemic. Further research is warranted to monitor the evolving mental health dynamics of Vietnamese migrants in Japan and elucidate their mental health characteristics.

## Conclusion

In this prospective cohort study on depression and anxiety, depressive symptoms among Vietnamese migrants declined from 2021 to 2022. Key findings underscore the importance of improving socioeconomic status and having access to someone with whom they can discuss their health as protective factors against mental health challenges. Employment and social support emerged as pivotal determinants of mental health among this population, underscoring the necessity for comprehensive support strategies addressing both economic vulnerabilities and social connectedness. It is essential to acknowledge the importance of bolstering the socioeconomic dimensions of migrant populations and establishing accessible mental health support systems, not only during the COVID-19 pandemic but also in future crises. By addressing these multifaceted challenges, policymakers and healthcare providers can more effectively meet the mental health needs of Vietnamese migrants in Japan and promote well-being within this community.

## Data Availability

The data that support the findings of this study are available from the corresponding author upon reasonable request.

## Abbreviations

PHQ-9: Patient Health Questionnaire-9
GAD-7: Generalized Anxiety Disorder 7-item
SD: Standard deviation
CI: Confidence interval
UPRC: Unstandardized partial regression coefficient

## Declarations

### Patient and public involvement

Patients and/or the public were not involved in the design, conduct, reporting, or dissemination plans of this research.
